# Using Surgical Microscope for Sclera Buckling and Transscleral Cryopexy: An Alternative Procedure of Treatment for Rhegmatogenous Retinal Detachment

**DOI:** 10.1155/2014/364961

**Published:** 2014-03-27

**Authors:** Liu-xue-ying Zhong, Yi Du, Wen Liu, Su-Ying Huang, Shao-chong Zhang

**Affiliations:** State Key Laboratory of Ophthalmology, Zhongshan Ophthalmic Center, Sun Yat-sen University, 54 South Xianlie Road, Guangzhou 510060, China

## Abstract

*Purpose.* To observe the long-term effectiveness of scleral buckling and transscleral cryopexy conducted under a surgical microscope in the treatment of uncomplicated rhegmatogenous retinal detachment.* Methods.* This was a retrospective analysis in a total of 227 consecutive patients (244 eyes) with uncomplicated rhegmatogenous retinal detachment (proliferative vitreoretinopathy ≤ C2). All patients underwent scleral buckling and transscleral cryopexy under a surgical microscope without using a binocular indirect ophthalmoscope or a contact lens.* Results.* After initial surgery, complete retinal reattachment was achieved in 226 eyes (92.6%), and retinal redetachment developed in 18 eyes (7.4%). The causes of retinal redetachment included presence of new breaks in eight eyes (44%), failure to completely seal the breaks in five eyes (28%), missed retinal breaks in four eyes (22%), and iatrogenic retinal breaks in one eye (6%). Scleral buckling surgery was performed again in 12 eyes (66%). Four eyes (22%) developed proliferative vitreoretinopathy and then were treated by vitrectomy. The sealing of retinal breaks and complete retinal reattachment were achieved in 241 eyes (98.8%).* Conclusion.* Probably because of clear visualization of retinal breaks and being controllable under a surgical microscope, the microsurgery of scleral buckling and transscleral cryopexy for uncomplicated retinal detachment exhibits advisable effectiveness.

## 1. Introduction

A goal persistently pursued by ophthalmologists is to be able to conveniently and clearly visualize the fundus during scleral buckling surgery. Only if the fundus is clearly visualized, the occurrence of intraoperative and postoperative complications can be reduced and the success rate for surgery can be improved.

During conventional scleral buckling surgery, localization and cryotherapy of retinal breaks are performed with a binocular indirect ophthalmoscope while drainage of subretinal fluid, intraocular gas injection, and suturing of bulbar conjunctiva are performed with the naked eye [1]. The greatest disadvantage of naked-eye observation is lack of elaborateness [2]. In 1971, Hilsdorf first performed retinal detachment surgery under a microscope with a three-mirror contact lens [3, 4]. Subsequently, many ophthalmologists used this method [3, 5]. However, as the cryoprobe used in this method can interfere with the observation with the three-mirror contact lens and the observed fundus image is inverted, a lot of operational inconveniences are caused [3, 5]. In clinical practice, we have developed a scleral buckling technique by which all surgical procedures are conducted under a surgical microscope. This technique does not require any contact lens, permits direct visualization of the location of retinal breaks, and has advantages of erect image, clear surgical field, elaborate and convenient operations, and good efficacy [6]. Some preliminary studies about this technique have been published in Chinese literature by our group [6–8]. Here, we reported a large case series of rhegmatogenous retinal detachment treated with this technique.

## 2. Patients and Methods

### 2.1. Patients

This study was approved by the institutional review board of the Zhongshan Ophthalmic Center and followed the tenets of the Declaration of Helsinki. All patients provided written consent.

Patients' eligibility criteria included surgeries performed by the corresponding author (WL) from December 2002 to September 2005, uncomplicated rhegmatogenous retinal detachment with proliferative vitreoretinopathy (PVR) ≤ C_2_ [9], transparent or mildly opaque refractive media that did not affect clear visualization of the fundus, and follow-up for at least 6 months. Exclusion criteria included a previous history of intraocular surgery (including open eye injury, cataract, retinal detachment, and vitreous surgery); cataract opacities > C_1_, N_1_, and P_1_ [10]; vitreous opacities > grade 2 [11] or PVR > C_2_; choroidal detachment, retinal breaks in the posterior pole, or giant retinal breaks (giant retinal breaks in the ora serrata were not included).

### 2.2. Surgical Treatment

#### 2.2.1. Preoperative Location of Retinal Breaks

The choice of surgical procedures was based on detailed mapping of the fundus changes according to the location of the retinal breaks using the three-mirror contact lens one day before operation [12]. Based on our experience, the areas viewed through a three-mirror contact lens can be converted as shown in Table 1.

After the retinal break(s) is pinpointed on the scleral surface, the location of the placement of the buckling material can be decided using the following formula:

(1) $$\begin{array}{c} B\pm \frac{1}{2}S\pm 1, \end{array}$$

where *B* is the posterior border of the break on the sclera posterior to the limbus, *S* the width of the buckling material, and 1 the place reserved for the scleral indentation. For example, if the posterior border of the break is 14 mm posterior to the limbus, the location of a 7 mm wide tire (style no. 276) on the scleral surface is

(2) $$\begin{array}{c} \text{anterior}\;\text{border}\;=14\;\text{mm}-7\;\text{mm}\times \frac{1}{2}-1\;\text{mm}=9.5\;\text{mm}\\ \text{posterior}\;\text{border}=14\;\text{mm}+7\;\text{mm}\times \frac{1}{2}+1\;\text{mm}=18.5\;\text{mm}. \end{array}$$

Thus, the span of the anterior-to-posterior mattress sutures is 9 mm. The 1/2 in the formula indicates that the 7 mm wide tire is divided between the anterior and posterior slope on average. If the break is 2.5 × 2.5 disk diameters, the anterior slope of the surgical buckle is sufficient to support it. The location of the buckling material on the scleral surface can be determined according to the experiential formula as long as the posterior border and the size of the breaks are estimated accurately preoperatively.

#### 2.2.2. Surgical Procedures

After 2% lidocaine and 0.75% bupivacaine were mixed equally, the retrobulbar anesthesia was induced. All procedures were completed under the surgical microscope (Zeiss Visu 200, Germany, and Leica 690, Heerbrugg, Switzerland).

**Figure 1:**
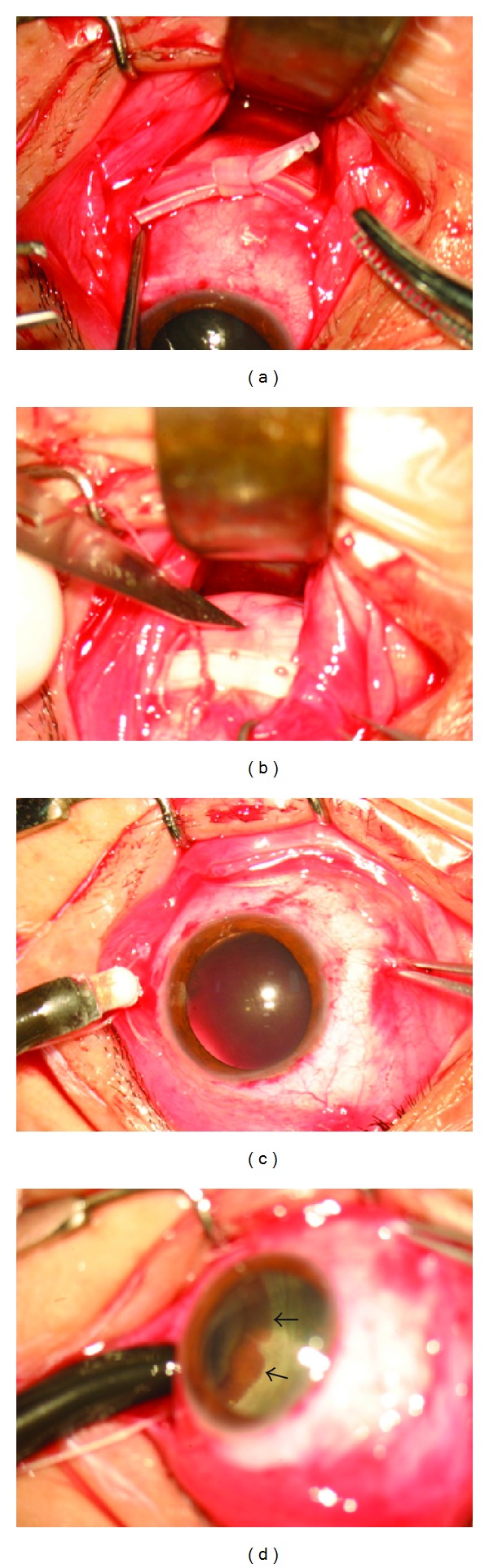
(a) The ends of the band are joined with the silicone sleeve. (b) Subretinal fluid is drained with an 11-gauge sharp-tipped knife. (c) Trying a cryotherapy probe and preparing cryotherapy. (d) The retinal dialysis is visualized through the scleral depression (arrow).

**Figure 2:**
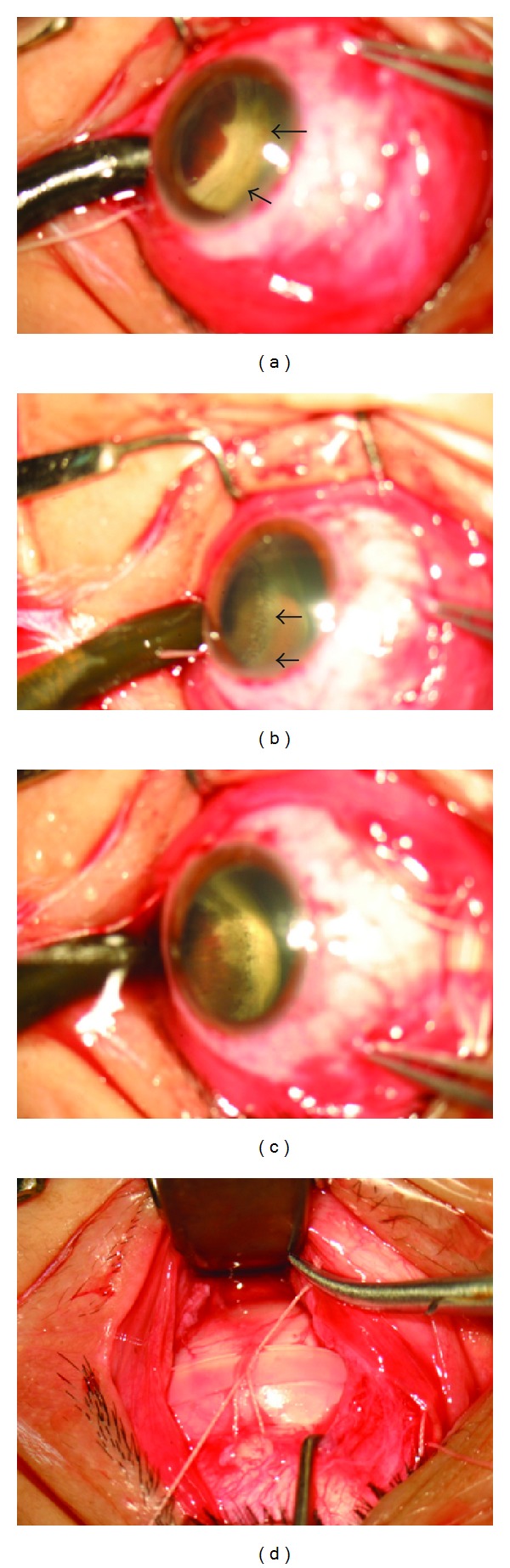
(a) The retina around the break gets white as cryotherapy is enough (arrow). (b) The cystic degeneration at the ora serrata is found (arrow). (c) The cryotherapy treatment is used for the cystic degeneration. (d) The preplaced suture for buckle is tightened.

**Figure 3:**
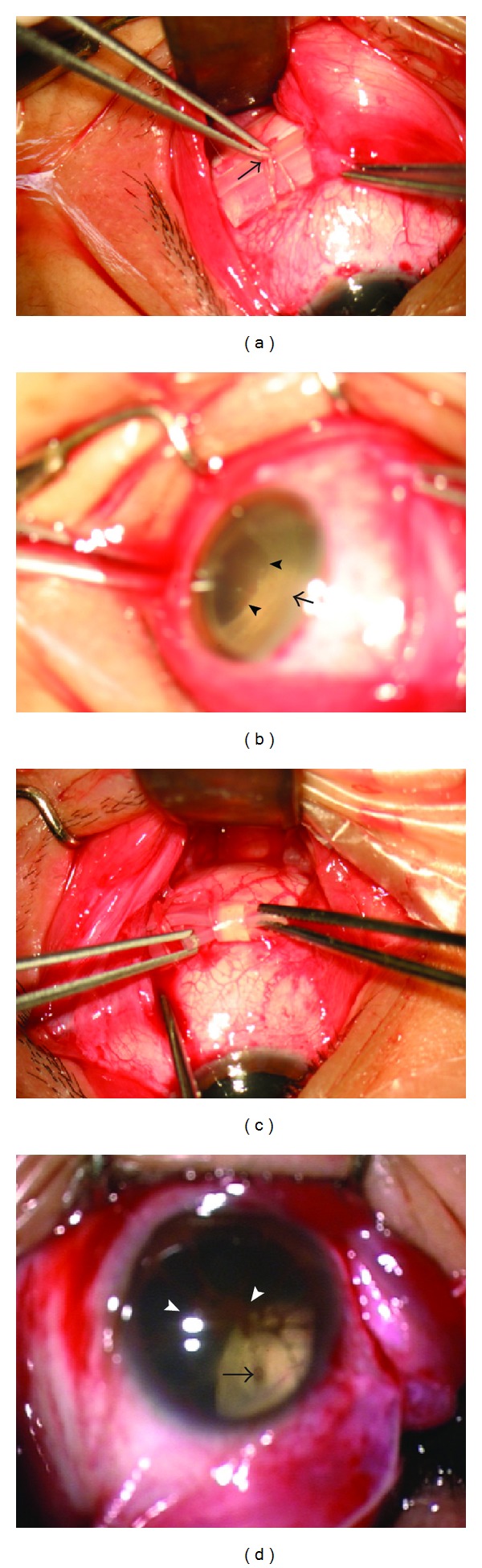
(a) The anterior-posterior middle (arrow) of the buckle is grasped to check up the location of the break on the anterior slope of the buckle. (b) The break (arrow head) is located on the anterior slope of the buckle (arrow). (c) The encircling band is tightened. (d) The picture presents intraoperative cryotherapy of the eye with the retinal hole and the residual membrane of the pupil (arrow head). The cryotherapy causes the retina whiting around the hole (arrow).

**Figure 4:**
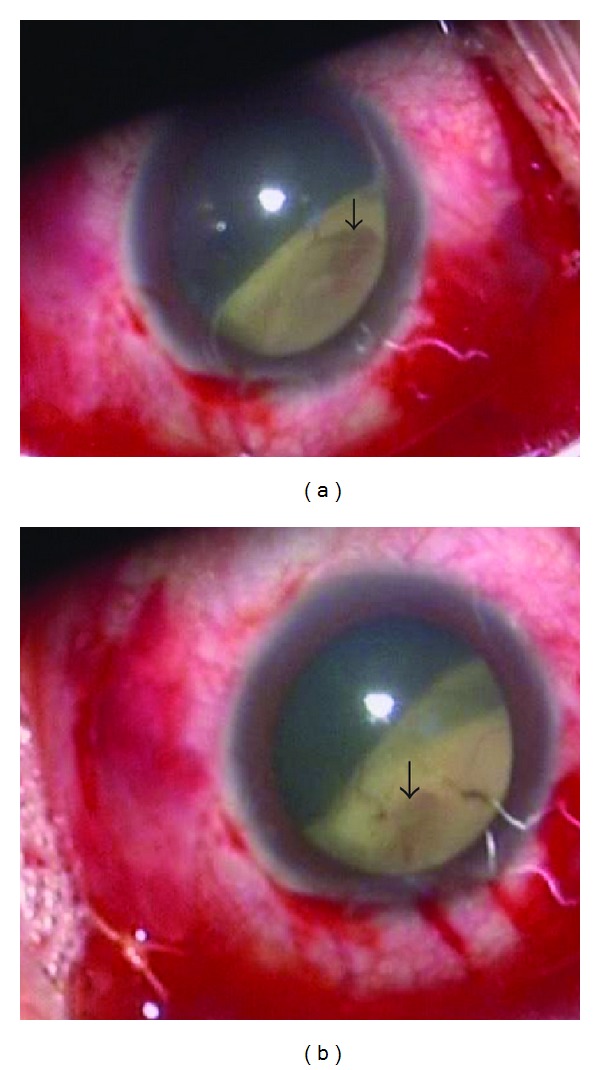
The break (arrow) that found to be too near to the posterior border of the buckle (a) and the surgeon adjusted the buckle backward to make the posterior border of the break (arrow) at about 2/3 disc diameter distance from the posterior border of the buckle (b).

The site for drainage of subretinal fluid was usually located 13–15 mm posterior to the corneal limbus. After preoperative examination, the drainage site was always selected under the superior and inferior rectus muscle or at the upper and lower margins of medial and lateral rectus muscles near the highest top of retinal detachment or near the break. The surgical assistant used the muscle hook and traction to expose the sclera under the rectus muscle. As the surgical assistant exposed the drainage site (depending on preoperative view and location), the surgeon held the sharp-tipped needle or sharp knife to penetrate the sclera at a 45-degree angle and release the subretinal fluid to soften the globe (Figure 1(b)). When insufficient subretinal fluid was drained or the intraocular pressure was still not lowered, about 0.1 to 0.3 mL of additional aqueous humor was drained by paracentesis of the anterior chamber to facilitate scleral depression. One end of the rectus muscle then was held with a tooth forceps to control and regulate the ocular position, and the cryoprobe was placed on the scleral surface (Figure 1(c)), on which the mattress sutures had already been placed, to depress the sclera and locate the retinal breaks. Retinal cryotherapy was started after the areas of the retinal breaks (Figures 1(d) and 2(a)) and degeneration were visualized (Figures 2(b) and 2(c)). Our pilot study found that a cryotherapeutic response could be visualized under the surgical microscope that progressed from choroidal congestion → retinal pigment epithelial whitening → retinal whitening→ice ball formation on the retinal surface. Thereafter, the retinal cryotherapy was stopped immediately as the retinal pigment epithelium or the retina whitened (Figure 2(a)). The episcleral silicone tire(s) was placed and the sutures were tied when cryotherapy was completed (Figure 2(d)). The location of the retinal breaks or the retinal degenerations was evaluated by scleral depression of the explants (Figures 3(a) and 3(b)). If the retinal break was flattened and located on the anterior slope of the scleral buckle, the operation was ended after the length of the encircling band (if any) was adjusted (generally shortened by less than 10 mm from 75 mm) (Figure 3(c)). If the retinal break was too far anterior or too far posterior, the location of the buckle was adjusted (Figures 4(a) and 4(b)). If the intraocular pressure was too low or there was a fish mouth-shaped break, an intravitreal injection of filtered air or expansible gas was considered. The final intraocular pressure was regulated to slightly higher than normal and the patient retained light perception. Short movie presenting the scleral buckling surgery under operating microscope is available at http://www.youtube.com/watch?v=IQ3aXyi6R_Y or http://my.tv.sohu.com/us/3431556/63381795.shtml.

Postoperative routine examinations were performed every day until the patient was discharged from hospital. Follow-up examinations were performed at 2 weeks, and 1, 3, 6, and 12 months postoperatively. The late reactions of the regions that received cryotherapy were classified into three grades: insufficient (no reaction), sufficient (pigment deposition and discoloration or appearance of a white color), and excessive (formation of an epiretinal membrane). If the areas treated with cryotherapy had two or more reactions, the more severe response was recorded.

#### 2.2.3. Statistical Analysis

The Chi-square test was used to compare preoperative and postoperative visual acuity. The data were processed on a computer with SPSS software 10.0 (SPSS, Chicago, IL, USA) for Windows. A *P* value of <0.05 was considered significant.

## 3. Results

A total of 281 cases (298 eyes) received scleral buckling and transscleral cryopexy under a surgical microscope. Fifty-four eyes were excluded from the study because of a previous history of eye injury (5 eyes), of cataract surgery (19 eyes), and of retinal detachment and vitreous surgery (12 eyes); choroidal detachment (7 eyes); cataract opacities (4 eyes); vitreous opacities > grade 2 (2 eyes); PVR > C2 (5 eyes).

The formal data analysis included 227 patients (154 males and 73 females, 244 eyes). These patients ranged in age from 5 to 76 years, with a median age of 38 years. Of 244 eyes, 131 were right and 113 were left. Partial corneal opacity in one eye and mild cataract in 25 eyes were observed. The number of breaks ranged from 1 to 8, with a median value of 2.0. The size of breaks ranged from 1 to 7 of disc diameter (DD) to the size of giant retinal breaks in the ora serrata. Ciliary epithelial tear in four eyes and the dialysis of ora serrata in 35 eyes were included. The breaks in 116 and 104 eyes had posterior borders within 11.5 mm (anterior to the equator of eyeball) and between 11.5 and 17.5 mm (at the equator of eyeball) posterior to the corneal limbus, respectively. Twenty-four eyes contained multiple breaks located anterior to the equator or at the equator. No patients had breaks located posterior to the equator. Of all eyes, 102 had breaks located in upper quadrants, 74 had breaks located in lower quadrants, 60 had breaks located in upper and lower quadrants, and 8 had breaks located exactly at 3 or 9 o'clock. The extent of retinal detachment varied from one quadrant to total detachment, with an average value of 6.6 clock hours. Lattice degeneration of retina was noted in 145 eyes, with an average extent of involvement of 2.94 clock hours. Subretinal demarcation line was observed in 64 eyes, with an average extent of involvement of 5 clock hours. All patients had grade 2 or less vitreous opacities [9]. PVR grading [7] showed that 75 eyes were of grade A, 164 eyes were of grade B, 3 eyes were of grade C_1_, and 2 eyes were of grade C_2_. Preoperative intraocular pressure levels ranged from 2.0 to 22.0 mmHg, with a median level of 11.1 mmHg.

### 3.1. Intraoperative Observations

Scleral buckling alone was performed in 88 eyes, while encircling scleral buckling was performed in 156 eyes. Drainage of subretinal fluid was conducted in 210 eyes, whereas 34 eyes underwent no drainage of subretinal fluid. Cryotherapy of the areas of retinal degeneration and breaks was performed successfully in all patients. The extent of cryotherapy ranged from 0.5 to 12 clock hours, with an average value of 3 clock hours. Mild opacity of refractive media did not affect the visualization and localization of retinal breaks as well as the observation of cryotherapeutic response (Figure 3(d)). Seventy-four preoperatively unobserved breaks, 71/74 of which were located at or around the ora serrata, were found in 30 eyes. At the end of the surgery, all breaks were confirmed to be located on the anterior slope of the buckle. Filtered air was injected in 126 eyes, and the injection volume ranged from 0.3 to 1.6 mL, with a median value of 0.64 mL. Perfluoropropane (C_3_F_8_, 100%) gas was injected in 34 eyes, and the injection volume ranged from 0.25 to 0.8 mL, with a median value of 0.48 mL. No gas was injected in 84 eyes. The duration of surgeries (including excision of pterygium in four eyes and strabismus correction in two eyes) ranged from 23 to 120 minutes, with a median value of 56 minutes.

### 3.2. Intraoperative and Postoperative Complications

Intraoperative complications included corneal epithelium scraped in 14 eyes (5.7%), penetration of the sclera by suture needle in 6 eyes (2.5%), iatrogenic retinal breaks in 6 eyes (2.5%; of which 4 (1.6%) developed the breaks due to fluid drainage and 2 (0.8%) due to penetration of the retina by suture needle), subretinal hemorrhage (occurred slightly and locally (≤2 DD) and did not enter the macular area) in 20 eyes (8.2%; of which 4 developed subretinal hemorrhage due to cryotherapy and the remaining due to drainage of subretinal fluid, but no case has hemorrhage going to submacular area), release of retinal pigment epithelium into the vitreous cavity via breaks in 26 eyes (10.7%) during cryotherapy and localization of retinal breaks, and disappearance of light perception in 5 eyes (2%) following gas injection (recovery after drainage of part of aqueous humor by anterior chamber paracentesis penetration).

Postoperative complications included fibrinous exudation in the anterior chamber in one eye (0.4%), conjunctival wound healing defect in two eyes (0.8%), anterior migration of silicone tire in one eye (0.4%), silicone explant infection in one eye (0.4%), choroidal detachment in four eyes (1.6%), macular epiretinal membrane formation in four eyes (1.6%), strabismus in one eye (0.4%), intraocular pressure elevation above 21 mmHg (mean: 32.2 mmHg; range: 22–72 mmHg; recovered to normal levels after prompt treatment) in 80 eyes (33%), and delayed subretinal fluid absorption in 53 eyes (22%; an average duration of 8 days, range 1–120 days).

### 3.3. Surgical Results

All patients were followed up for at least 6 months, with an average duration of 19.5 months. After initial surgery, retinal reattachment was achieved in 226 eyes (92.6%), and retinal redetachment occurred in 18 eyes (7.4%). The causes of retinal redetachment included occurrence of new breaks in eight eyes (44%), failure to completely seal breaks in five eyes (28%), missed retinal breaks in four eyes (22%), and iatrogenic retinal breaks caused by fluid drainage in one eye (6%). Scleral buckling surgery was performed again in 12 eyes, of which one eye did not achieve retinal reattachment. Four eyes (22%) developed PVR and needed vitreous surgery. After first vitreous surgery, retinal reattachment was achieved in all eyes. Treatment was discontinued in two eyes due to patient abandonment. Finally, sealing of retinal breaks and complete retinal reattachment were achieved in 241 eyes (98.8%). At the last follow-up, 22 eyes had a best-corrected visual acuity below 0.05, 82 between 0.05 and 0.3, 69 between 0.4 and 0.9, and 71 greater than or equal to 1.0. A significant difference between preoperative and postoperative best-corrected visual acuity was noted (Table 2; *P* = 0.0005, *χ* ^2^ = 84.439).

## 4. Discussion

Based on our experience, there is no difference between the indications for scleral buckling surgery conducted under a surgical microscope and those for conventional scleral buckling surgery. Contrasted to using direct ophthalmoscope or binocular indirect ophthalmoscope, it is an alternative surgical method of using surgical microscope to treat retinal breaks. The core technique is the use of a surgical microscope to perform scleral buckling surgery, directly visualize retinal breaks or lesions, and then perform transscleral cryopexy without using any contact lens. Preoperative examination with a three-mirror contact lens is used to localize the areas of retinal breaks and degeneration to guide scleral buckling and cryotherapy of retinal lesions intraoperatively. And a formula is used to preoperatively estimate the location of silicone tire on the scleral surface. Moreover, the methods for intraoperative scleral exposure, subretinal fluid drainage, and retinal cryotherapy have been improved. Even more, the accurate location of the buckling is evaluated by pushing the silicone tire intraoperatively [6, 7].

The intraoperative complications encountered in this study included corneal epithelial edema and defects, flocculent exudation in anterior chamber, penetration of the sclera by suture needle, fluid drainage-associated complications, release of retinal pigment epithelium into the vitreous cavity, and temporary disappearance of light perception following gas injection, while postoperative complications included corneal epithelial exfoliation, delayed subretinal fluid absorption, temporary glaucoma, choroidal detachment, gas injection-related cataract, macular epiretinal membrane formation, and retinal redetachment. The manifestations of these complications and the mechanisms underlying their occurrence were similar to those observed in conventional scleral buckling surgery performed with a binocular indirect ophthalmoscope [13]. After prompt treatment, these complications were uneventful. However, it is worth to note that the incidence of postoperative choroidal detachment observed in this study was very low (only 1.6%). This may be due to the fact that double-handed operation is used in this technique, which can effectively maintain constant intraocular pressure during the surgery and thereby reduce the occurrence of postoperative choroidal detachment.

The main shortcoming of this surgical method is that the retina at the posterior pole cannot be visualized. However, we believe whether intraoperative retinal reattachment of the posterior pole or not has no decisive effect on the success of surgery. If effective sealing of breaks is achieved during the operation, residual subretinal fluid can be absorbed spontaneously. On the other hand, if the breaks are located at the posterior pole, it is beyond the reach of scleral buckling surgery, and vitreous surgery has to be performed. Thus, scleral buckling and transscleral cryopexy performed under a surgical microscope are a very effective method. The advantages and disadvantages of scleral buckling surgery between using indirect ophthalmoscope and using microscopy have been summarized in Table 3.

A limitation of this study was that it had no a comparison group to show whether retinal surgery under surgical microscope had better efficacy, since the surgeon (WL) does not perform surgery using indirect ophthalmoscope in clinical practice. However, this large case series suggested this procedure is an effective and safe method, and this is an alternative choose for other retinal surgeons.

In summary, use of the surgical microscopy for scleral buckling and transscleral cryopexy may lead to a high rate of anatomical and functional recovery in the treatment of uncomplicated rhegmatogenous retinal detachment. This should be contributed by multiple advantages of this surgical method, including simpleness, erect images observed intraoperatively, clear visualization of retinal breaks, and being easy to learn and use. We believe that scleral buckling and transscleral cryopexy performed under a surgical microscope have the potential to become one of the select surgeries for uncomplicated retinal detachment.

## Figures and Tables

**Table 1:** Areas viewed through three-mirror contact lens (modified from Schepens [12]).

	Lens I	Lens II	Lens III	Lens IV
	(Contact lens)	(Lens)	(Lens)	(Lens)
Areas viewed	30 degrees around the macula	From 30 degrees to equatorial fundus	Peripheral fundus	Extreme fundus periphery
Areas posterior to the limbus	The posterior pole of the fundus	13–17 mm* (chord length)	10–15 mm* (chord length)	9 mm* (chord length)

*An additional 0.5 millimeter should be added for each additional diopters of −3.0 in eyes with myopia.

**Table 2:** Comparison of preoperative and postoperative visual acuity in 244 eyes with rhegmatogenous retinal detachment.

Best-corrected visual acuity	<0.05	0.05~0.3	0.4~0.9	≥1.0	*P* value*
Before surgery	89	103	31	21	0.0005
After surgery	22	82	69	71	

*Chi-square test.

**Table 3:** Comparison of surgery using surgical microscope versus using indirect ophthalmoscope for scleral buckling and transscleral cryopexy.

	Using surgical microscope	Using indirect ophthalmoscope
Advantages	Continuous zoom (no need for accommodation of naked eye of operator)	Wide view
	Erect image	Stereopsis
	Easy to suture accurately	Posterior pole can be seen easily
	Pre-equatorial and peripheral retina can be seen easily	

Disadvantages	Need to press peripheral retina strongly	Inverted image
	Impossible to see posterior pole	Hard to see details of retina
	Narrow view	Need for accommodation of naked eye of operator
